# Generalizability of Low‐Voltage Zone‐Based Risk Scores After Atrial Fibrillation Ablation

**DOI:** 10.1002/clc.70365

**Published:** 2026-06-02

**Authors:** Laiba Anwar, Aina Gul, Javeria Kashaf Zain, Muhammad Faizan Khan, Syed Huzaifa Khan

**Affiliations:** ^1^ Nowshera Medical College Nowshera Pakistan; ^2^ Khyber Medical University Peshawar Pakistan

To the Editor,

We read with great interest the recent article by Han et al. [1], in which the authors proposed a novel multiparametric score integrating low‐voltage zone (LVZ) mapping with readily available biomarkers to predict atrial fibrillation recurrence following catheter ablation. The effort to combine electrophysiological substrate characterization with clinical biomarkers is timely and innovative. However, given that LVZ assessment constitutes a central component of the proposed model, its clinical interpretation warrants careful consideration. In particular, the extent to which LVZ‐based risk stratification can be generalized across centers with differing mapping techniques, operator practices, and ablation strategies remains uncertain. We therefore wish to highlight several methodological and interpretive considerations that may influence the model's external validity and clinical applicability.

A major limitation lies in the implicit presentation of the proposed AF‐recurrence score as broadly generalizable and clinically applicable, despite its strongest determinant, left atrial LVZ extent, being highly operator, protocol, and central dependent. A reasonable reader may therefore infer that the score can be applied in routine practice across centers. However, atrial voltage mapping is known to vary substantially with mapping catheter characteristics, electrode configuration, point density, and acquisition technique, leading to mark difference in quantified LVZ burden [2]. Additionally, LVZ assessment is rhythm‐dependent, with sinus rhythm and atrial fibrillation mapping producing materially different substrate distributions [3]. The absence of inter‐center reproducibility testing renders the score context‐specific rather than universally transferable, weakening its external validity.

Several additional considerations further contextualize the interpretations of the model. First, the retrospective single‐center design introduces the possibility of selection bias, a recognized limitation of prognostic model development studies [4]. Moreover, the model underwent internal validation only; without external multicenter validation, its predictive performance across populations with different ablation strategies and patient characteristics cannot be assumed [5]. In addition, atrial fibrillation recurrence may be asymptomatic, and follow‐up strategies relying on scheduled electrocardiography rather than continuous rhythm monitoring may underestimate recurrence rates, influencing outcome classification [6]. While secondary, these factors may further contribute to variability in model performance when applied in broader clinical contexts.

We commend the authors for their integrative and clinically motivated approach to risk prediction following atrial fibrillation ablation.

Nevertheless, given the central role of LVZ assessment within proposed score, its translation into routine clinical practice may be constrained by variability in substrate mapping and procedural techniques across centers. Clarifying these boundaries through prospective, multicenter studies with standardized mapping protocols and rigorous rhythm surveillance will be essential to establish the robustness and generalizability of this model and to support its safe incorporation into clinical decision‐making.

## Author Contributions

All authors have read and approved the final manuscript.

## Funding

The authors have nothing to report.

## Ethics Statement

The authors have nothing to report.

## Consent

The authors have nothing to report.

## Conflicts of Interest

The authors declare no conflicts of interest.

## Data Availability

The authors have nothing to report.
